# Machine Learning Using a Single-Lead ECG to Identify Patients With Atrial Fibrillation-Induced Heart Failure

**DOI:** 10.3389/fcvm.2022.812719

**Published:** 2022-02-28

**Authors:** Giorgio Luongo, Felix Rees, Deborah Nairn, Massimo W. Rivolta, Olaf Dössel, Roberto Sassi, Christoph Ahlgrim, Louisa Mayer, Franz-Josef Neumann, Thomas Arentz, Amir Jadidi, Axel Loewe, Björn Müller-Edenborn

**Affiliations:** ^1^Institute of Biomedical Engineering (IBT), Karlsruhe Institute of Technology (KIT), Karlsruhe, Germany; ^2^Division of Cardiology and Angiology II, University Heart Center Freiburg-Bad Krozingen, Bad Krozingen, Germany; ^3^Dipartimento di Informatica, Università degli Studi di Milano, Milan, Italy

**Keywords:** atrial fibrillation, heart failure, machine learning, ECG, RR intervals, diagnostic tool

## Abstract

**Aims:**

Atrial fibrillation (AF) and heart failure often co-exist. Early identification of AF patients at risk for AF-induced heart failure (AF-HF) is desirable to reduce both morbidity and mortality as well as health care costs. We aimed to leverage the characteristics of beat-to-beat-patterns in AF to prospectively discriminate AF patients with and without AF-HF.

**Methods:**

A dataset of 10,234 5-min length RR-interval time series derived from 26 AF-HF patients and 26 control patients was extracted from single-lead Holter-ECGs. A total of 14 features were extracted, and the most informative features were selected. Then, a decision tree classifier with 5-fold cross-validation was trained, validated, and tested on the dataset randomly split. The derived algorithm was then tested on 2,261 5-min segments from six AF-HF and six control patients and validated for various time segments.

**Results:**

The algorithm based on the spectral entropy of the RR-intervals, the mean value of the relative RR-interval, and the root mean square of successive differences of the relative RR-interval yielded an accuracy of 73.5%, specificity of 91.4%, sensitivity of 64.7%, and PPV of 87.0% to correctly stratify segments to AF-HF. Considering the majority vote of the segments of each patient, 10/12 patients (83.33%) were correctly classified.

**Conclusion:**

Beat-to-beat-analysis using a machine learning classifier identifies patients with AF-induced heart failure with clinically relevant diagnostic properties. Application of this algorithm in routine care may improve early identification of patients at risk for AF-induced cardiomyopathy and improve the yield of targeted clinical follow-up.

## Introduction

Atrial fibrillation (AF) is the most common arrhythmia in human kind, affecting approximately eight million patients in the European Union (1). Fibrillatory activity in the atria not only promotes atrial thrombus formation and systemic thromboembolism, but also leads to irregular and often rapid ventricular activation.

AF and heart failure share many common risk factors, predispose to each other, and often coexist (2). AF can occur concomitantly with heart failure without causative relation, and restoration of sinus rhythm in these patients results in only modest improvements of left ventricular systolic dysfunction (LVSD). In a potentially large subset of patients with AF and heart failure however, sinus rhythm restoration leads to drastic improvements or normalization of LVSD (3–6) within days to weeks.

It is currently not fully understood why certain patients develop severe heart failure symptoms and LVSD during AF (AF-induced heart failure; AF-HF). Current guidelines emphasize the importance of AF in this context, and recommend routine clinical follow-up in AF patients to recognize cardiac deterioration early (1). The most established modality to detect heart failure in this patient group remains echocardiography, unfortunately including all its limitations with regard to the equipment and training of the examiner that is required. Given the ever-increasing prevalence of AF in the European population, easily applicable screening tools to identify patients at risk are desirable to tailor patient care and reduce costs for health care systems.

Machine learning-based algorithms are an emerging tool in diagnosis and risk prediction and have shown promising results in the field of cardiology (7). A feature-based machine learning algorithm can lead to a clear interpretation of the results as clinical algorithms do. However, to develop a performant feature-based classifier a careful selection of features that have been recognized as relevant in the analysis of heart rhythms should be made.

We hypothesize that specific patterns of ventricular beat-to-beat variations and arrhythmia characteristics in AF are associated with the clinical phenotype of AF-HF, potentially enabling early prediction of AF-patients at risk to develop heart failure.

In the following manuscript, we show the methods and procedures used to implement a classification between patients with AF-HF and control group patients (in AF but without risk of developing heart failure) using 5-min RR signals acquired during daylight hours (from 8 a.m. to 10 p.m.). In addition, an analysis of the influence of the circadian cycle on classification performance was performed.

## Methods

### Study Protocol

This prospective observational study was approved by the local institutional review board, and patients gave informed consent. Inclusion criteria were persistent (lasting 7 days to 12 months) or long-persistent (lasting >12 months) AF at study screening, absence of left- or right-sided significant valvulopathies (moderate or severe), and absence of relevant coronary artery disease as evidenced using coronary angiography or non-invasive imaging within 12 months of screening. Patients younger than 18 years and those with a history of ischemic heart disease requiring revascularization with or without myocardial infarction were excluded.

All study participants underwent standard 12-lead ECG, 24 h Holter single-lead ECG, and transthoracic echocardiography within 24 h from study inclusion (Supplementary Figure 3). Details on echocardiographic assessment of LVEF are provided in the Supplementary Material. Patients with LVEF >50% in AF were considered as control group. Patients with an initial LVEF ≤ 40% in AF were scheduled for electro-cardioversion on the next working day and underwent additional clinical follow-up including repeat echocardiography at day 40. As the current study focuses on AF-induced heart failure, only patients who experienced an absolute improvement of LVEF of 15% or more within 40 days in sinus rhythm remained in the study for further analysis (6). Patients who either experienced AF-recurrence within 40 days from cardioversion or who experienced an improvement in LVEF of <15% despite sinus rhythm were excluded from this study (*n* = 6 and *n* = 3, respectively).

The primary endpoint was the determination and validation of an algorithm to identify AF-HF patients from 5-min Holter ECG segments recorded during daytime (8 a.m. to 10 p.m.). Secondary endpoints were the performance of the feature set for nighttime (10 p.m. to 8 a.m.) and full-day times (8 a.m. to 8 a.m.).

### ECG Data Extraction

Consecutive RR-intervals (RR) were extracted from the single-lead 24 h Holter ECG raw data set using the Cardioday software (Getemed Medizintechnik, Teltow, Germany) with a 128 Hz-sampling rate. Prior to extraction, the complete data set was manually screened, and noise or artifacts were excluded by two senior electrocardiogram-analysts. Relative RR-intervals (relRR) were calculated as a percentage of the current RR-interval N with respect to the previous RR-interval N-1. Based on the conventional short-term recording standards (8), intervals were grouped in segments of 5 min each, resulting in a total of 10,234 segments. Two-thousand one hundred-four AF-HF and 2,301 control group daytime segments (recorded between 8 a.m. to 10 p.m.) were analyzed. Moreover, a full-day set and a night set (from 10 p.m. to 8 a.m.) were analyzed to check the circadian differences in performance. The full-day set comprised 5,266 segments in the AF-HF group, and 4,968 segments in the control group group, whereas the night set comprised 3,162 AF-HF, and 2,667 control group segments.

### Feature Extraction

Fourteen features were extracted from the signals (8 from RR, and 6 from relRR series) using several clinical heart rate variability (HRV), and advanced biosignal processing parameters to derive information regarding the regularity and complexity of the time series: the mean RR and mean relRR intervals ($\bar{RR}$ and $\bar{relRR}$), time between all adjacent heartbeats; the standard deviation of the RR and relRR intervals (*SDRR* and *SDRR*_*rel*_) to measure how these intervals vary over time; the root mean square of successive differences between heartbeats (*RMSSD*_*RR*_ and *RMSSD*_*relRR*_) reflecting the beat-to-beat variance in heart rate (HR) (9); the deceleration capacity (*DC*) providing a measure of cardiac vagal modulation; the deceleration reserve (*DR*) to measure the balance between deceleration and acceleration capacity emphasizing asymmetric growing, decaying HR trends, and non-stationarity (10); the Shannon entropy of the RR and relRR series (*ShanEn*_*RR*_ and *ShanEn*_*relRR*_) to assess the complexity of the signals based on information theory; the sample entropy (*SampEn*_*RR*_ and *SampEn*_*relRR*_) measuring the complexity of the time series (9); and spectral entropy (*SpecEn*_*RR*_ and *SpecEn*_*relRR*_) indicating the spectral complexity of these time series (11). More information regarding the feature extraction methods is provided in the Supplementary Material.

### Feature Selection and Evaluation

A greedy forward selection technique was implemented to select the optimal feature set out of the 14. This algorithm started with an empty feature set and added, in each iteration, the feature which led to the highest classification performance increase assessed using the accuracy of a decision tree classifier (see Section Feature Selection and Evaluation for details about the classifier). The algorithm stopped when performance based on the validation set (subset of data utilized to tune the algorithm's parameters) could not be further increased. Candidate features to be added to the set were only added if the correlation coefficient with any of the already included features was <0.6. The correlation threshold was optimized looking for the best compromise between redundant information and physiological explanation only deleting the features that have similar values' distributions and that are expression of the same physiological behavior.

Shapley calculation was implemented to analyze a posteriori the importance of the features selected for classification once the model was trained (12). The Shapley calculation was run 1,000 times with random samples to calculate the standard deviation (SD).

### Machine-Learning Classification

A decision tree classifier was implemented for binary classification (AF-HF vs. control group) for the daytime set. The decision tree algorithm was selected due to its simplicity and explainability. The decision tree was trained, and applied using the MATLAB functions fitctree, and predict, respectively. Similar analyses were performed using different machine learning algorithms and with different segment lengths (see Supplementary Material).

**Figure 1:**
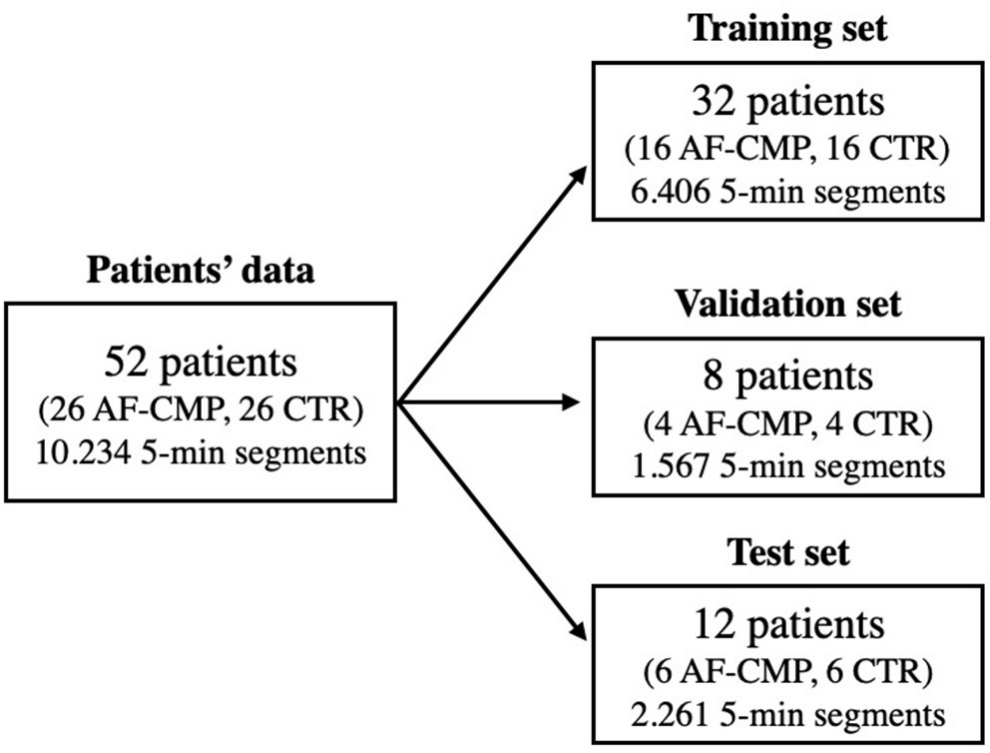
Flow chart showing the dataset division of the 52 patients' signals into training, validation, and test sets, respectively. The number of all 5-min segments acquired from the patients is reported as well. Control group (CTR).

The multi-feature classification was performed with the feature set selected as described in Section Feature Selection and Evaluation. Five-fold cross-validation was performed by randomly dividing the dataset into a training set, validation set, and test set with 32, 8, and 12 patients in each set, respectively (Figure 1). Training and validation sets were recalculated at each iteration while the test set was excluded and used only once on the final classifier. The final classifier was obtained by re-training it with all the data (training + validation sets). This approach allowed us not to include RR series from the same patient in different sets, and not to use the test set during algorithm development, thus avoiding overfitting on the data. The classes were always balanced between the two groups, however for shrewdness the Prior model parameter in the MATLAB fitctree function was set to uniform. Sensitivity, specificity, and positive predictive value (PPV) were calculated considering the AF-HF group as positive, and the control group group as negative. The choice of these performance metrics is motivated by their extensive use in the clinical and biomedical engineering fields regarding machine learning approaches applied to biomedicine and being considered basic concepts of this field.

Moreover, a decision tree single-feature classification was implemented with each individual feature of the set to compare their individual classification power against that of the multi-feature classifier.

Regarding the full-day set, and the nighttime set, we first computed classifiers using the feature set extracted for the daytime set. Then, we implemented two new classifiers where the feature sets were optimized for the full-day, and nighttime set by greedy selection (see Section Feature Selection and Evaluation), respectively.

### Statistical Analysis

Statistical analysis was performed using SPSS version 25.0 for macOS (IBM Corporation, Armonk, New York), or GraphPad Prism version 8 for macOS (GraphPad Software, La Jolla, California). Normally distributed data are expressed as mean ± SD, skewed data are expressed as median (interquartile range). Intergroup comparisons were performed using student's *t*-test, or Mann-Whitney-test depending on normality.

Classifier performance was evaluated using accuracy (ACC), sensitivity, specificity, and PPV. Accuracy was also calculated for each individual patient in the test set (ACCi, with i as test set patient ID, **Table 2**) by counting how many segments belonging to the same patient were correctly classified with respect to their total number.

The comparison between the feature distributions, and AF-HF, and control group groups was done using the Wilcoxon rank sum test (one-tailed, *p* < 0.05 considered significant).

## Results

### Patient Characteristics

A total of 52 patients (26 with AF-HF and 26 control group) were included in the study. All patients were in persistent or long-persistent AF at study inclusion. Descriptive data of study participants are given in Table 1. Patients with AF-HF had higher NYHA stages, higher average heart rates, and were more often on ACE inhibitors and aldosterone antagonists, as well as on antiarrhythmics.

**Table 1:** Descriptive patient characteristics.

	**All**	**AF-HF**	**CTR**	
	***n* = 52**	***n* = 26**	***n* = 26**	***p*-value**
Age (years)	68.3 (11.7)	70.48 (11.83)	66.3 (11.54)	0.204
Male sex	26 (50)	18 (69.2)	17 (65.5)	1.000
BMI (kg/m^2^)	29.1 (4.9)	29.59 (5.84)	28.74 (4.03)	0.416
Systolic blood pressure (mmHg)	135.7 (21.1)	132.4 (22.66)	139 (19.25)	0.264
Diastolic blood pressure (mmHg)	87.42 (13.1)	85.81 (13.77)	89.04 (12.49)	0.380
**NYHA stages**				<0.001
NYHA I	8 (15)	1 (3.8)	7 (26.9)	
NYHA II	10 (19)	2 (7.7)	8 (30.8)	
NYHA III	19 (36.5)	9 (34.6)	10 (38.5)	
NYHA IV	15 (28.8)	14 (53.8)	1 (3.8)	
Diabetes	7 (13.5)	0 (0)	7 (26.9)	0.01
Hypertension	35 (67)	17 (65.4)	18 (69.2)	1.000
Hyperlipidemia	26 (50)	12 (46.2)	14 (53.8)	0.782
**Medications**				
ß blocker	37 (71)	19 (73.1)	18 (69.2)	1.000
ACE inhibitors	22 (42.3)	16 (61.5)	6 (23.1)	0.011
ATRA	10 (19.2)	4 (84.6)	6 (76.9)	0.726
Mineralcorticoid receptor blocker	14 (26.9)	12 (46.2)	2 (7.7)	0.004
Diuretics	21 (40.4)	13 (50)	8 (30.8)	0.160
Digoxin	2 (3.8)	0 (0)	2 (7.7)	0.490
Antiarrhythmics (class 1c and class 3 cumulative)	19 (36.5)	16 (61.5)	3 (11.5)	<0.001
**Echocardiography**				
LVEF	44.8 (15.9)	29.25 (6.78)	59.15 (2.64)	<0.001
LVESD (mm)	39 (9.9)	45.67 (8.78)	31.9 (4.75)	0.004
LVEDD (mm)	52 (7.0)	55.48 (7.80)	49.92 (5.03)	<0.001
LAD (mm)	45 (6.4)	48.54 (4.86)	42.38 (6.4)	<0.001
LAV (ml)	96.4 (27.4)	106.84 (18.13)	75.6 (31.17)	0.002
LAVI (ml/kg/BW)	49 (9.6)	51.94 (7.35)	42.38 (11.47)	0.017
**ECG**				
Resting heart rate in 12-lead ECG	93.4 (24.2)	104 (23.9)	82.6 (19.5)	0.001
Mean heart rate in 24 h- ECG	85.3 (17.2)	91.6 (16.6)	78.7 (15.4)	0.006
QRS width (ms)	93.3 (16.0)	95.1 (19.2)	91.4 (12.2)	0.407

*Control group (CTR), body mass index (BMI), New York Heart Association (NYHA), angiotensin converting enzyme (ACE), angiotensin type 1 receptor antagonist (ATRA), left ventricular ejection fraction (LVEF), left ventricular end-systolic diameter (LVESD), left ventricular end-diastolic diameter (LVEDD), left atrial diameter (LAD), left atrial volume (LAV), left atrial volume index (LAVI). Values are given as mean (± standard deviation) or number (%)*.

### Feature Selection and Algorithm Performance to Detect Atrial Fibrillation-Induced Heart Failure

**Figure 2:**
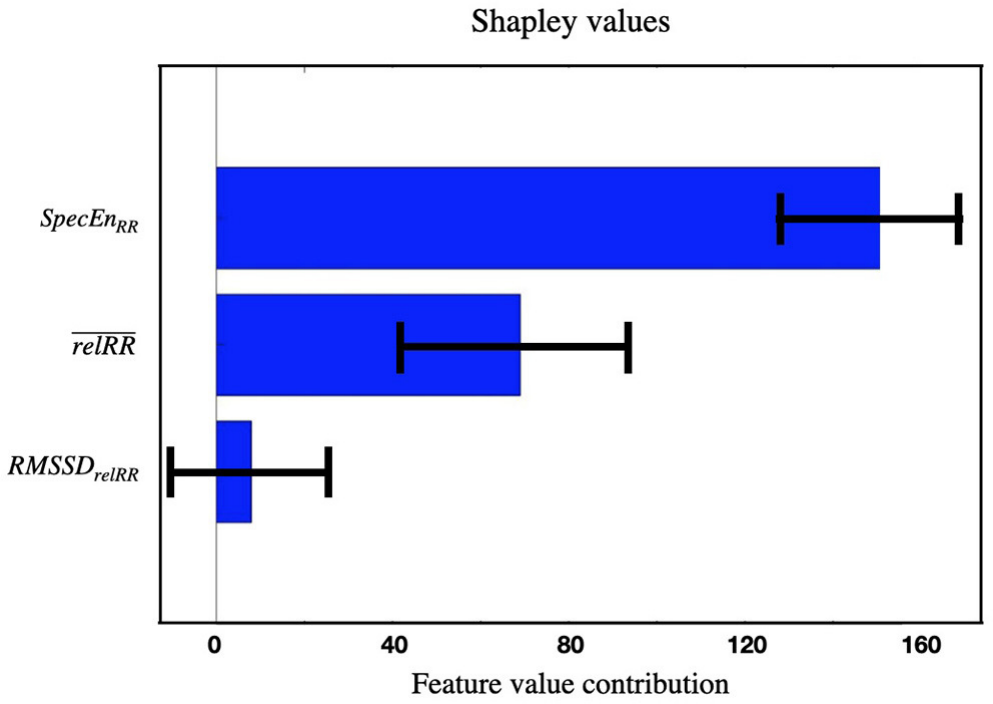
Shapley feature importance calculation on the three features selected for the daytime binary classification AF-HF vs. control group. The shapley calculation was run 1,000 times with random samples to calculate the SD (error bars in the plot).

Splitting the longitudinal Holter ECG data into intervals of 5 min each and selecting only segments recorded during daytime (8 a.m. to 10 p.m.) resulted in a total of 4,405 segments (2,104 segments for AF-HF and 2,301 segments for control group). Greedy forward selection on these data led to a feature set composed of three out of the 14 features extracted in total: $SpecEn_{RR},\;\bar{relRR}$, and *RMSSD*_*relRR*_. Evaluation of the relative contribution of each feature to the overall classification demonstrated the highest contribution for *SpecEn*_*RR*_, followed by $\bar{relRR}$ and *RMSSD*_*relRR*_ (Figure 2). In the Supplementary Material the *SpecEn*_*RR*_ values' distribution is shown for the control group and AF-HF groups.

**Figure 3:**
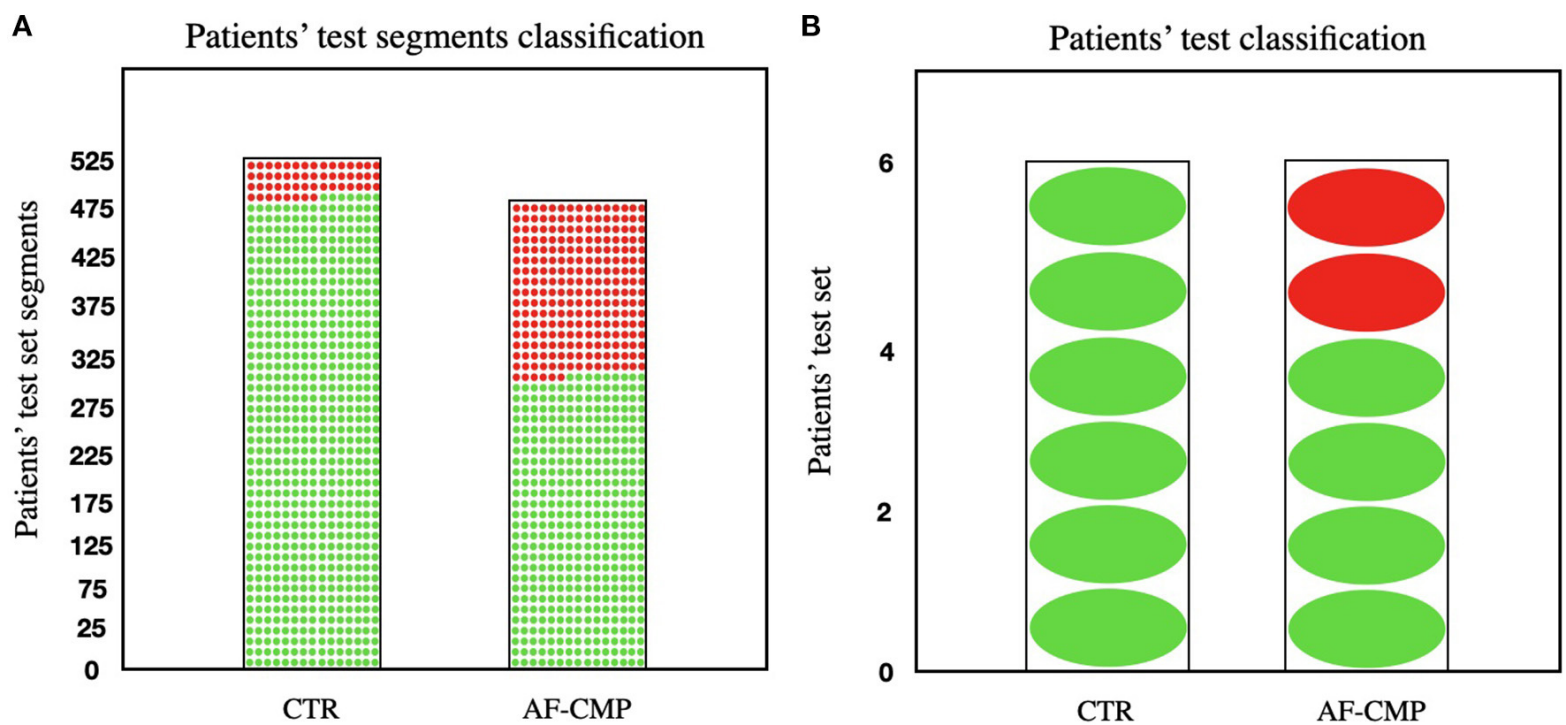
**(A)** Visual representation of the number of segments in the test set that were correctly classified for both control group and AF-HF groups (91.4% and 64.7% of the segments correctly classified for each group, respectively). **(B)** Visual representation of the number of individual patients in the test set that were correctly classified for both control group and AF-HF groups (100% and 83.3% of the patients correctly classified for each group, respectively). The red dots represent segments/patients misclassified; the green dots represent segments/patients correctly classified. Control group (CTR).

Application of the decision tree classifier with this feature set on the patients in the test set (475 AF-HF, and 525 control group 5-min segments from six AF-HF, and six control group patients, respectively) yielded an overall accuracy to correctly assign a given 5-min segment to AF-HF or control group of 73.5%, with a specificity of 91.4%, sensitivity of 64.7%, and PPV of 87.0% (Figure 3). When applying a 50% threshold on the fraction of segments correctly classified for a given patient, 10 out 12 patients (83.3%) were correctly assigned to AF-HF or control group (6/6 patients in the control group group and 4/6 in the AF-HF group; Figure 3). The accuracy achieved for each individual patient in the daytime test set is given in Table 2. Similar results have been achieved using other machine learning algorithms (see Supplementary Material).

**Table 2:** Number of segments and accuracy for each individual patient in the test set (%) for the daytime dataset.

**Test set patient ID**	**No. of segments**	**Class**	**ACC_P_**i**_**
1	72	AF-HF	76.19
2	77	AF-HF	56.10
3	85	AF-HF	57.14
4	64	AF-HF	81.43
5	78	AF-HF	43.80
6	99	AF-HF	17.81
7	85	CTR	85.39
8	88	CTR	92.13
9	112	CTR	96.34
10	78	CTR	97.06
11	96	CTR	93.59
12	66	CTR	85.19

*Patients who were correctly classified over all segments (ACC_Pi > 50%) are highlighted in green. Patients who got misclassified over all segments (ACC_P_i_ <50%) are highlighted in red. Control group (CTR)*.

### Circadian Performance Differences on the Classification

The decision tree classifiers derived from Holter recordings during daytime as described above ($\bar{relRR}$, *RMSSD*_*relRR*_, and *SpecEn*_*RR*_) yielded an accuracy of only 56.5% when applied on all available 5-min-segments (recorded between 8 a.m. and 8 a.m. the next day, *n* = 2,261), and 49.3% for segments recorded during nighttime (10 p.m. to 8 a.m., *n* = 1,261).

An optimized feature set for all segments (recorded between 8 a.m. and 8 a.m. the next day) based on the greedy forward selection was composed of 10 features out of the 14 extracted (*ShanEn*_*RR*_, *RMSSD*_*relRR*_, *ShanEn*_*relRR*_, $\bar{RR}$, *DR*, *SampEn*_*RR*_, *SpecEn*_*RR*_, *DC*, *SpecEn*_*relRR*_, and *SDRR*). The classifier retrained on this optimized feature set yielded an improved accuracy on all segments of the test set of 60.5%, specificity, and sensitivity of 64.2%, and 57.3%, respectively, and a PPV of 62.2%. With respect to the total number of segments for each patient, 10/12 patients (83.3%) were classified correctly (5/6 control group patients, and 5/6 AF-HF patients, table in the Supplementary Material).

**Figure 4:**
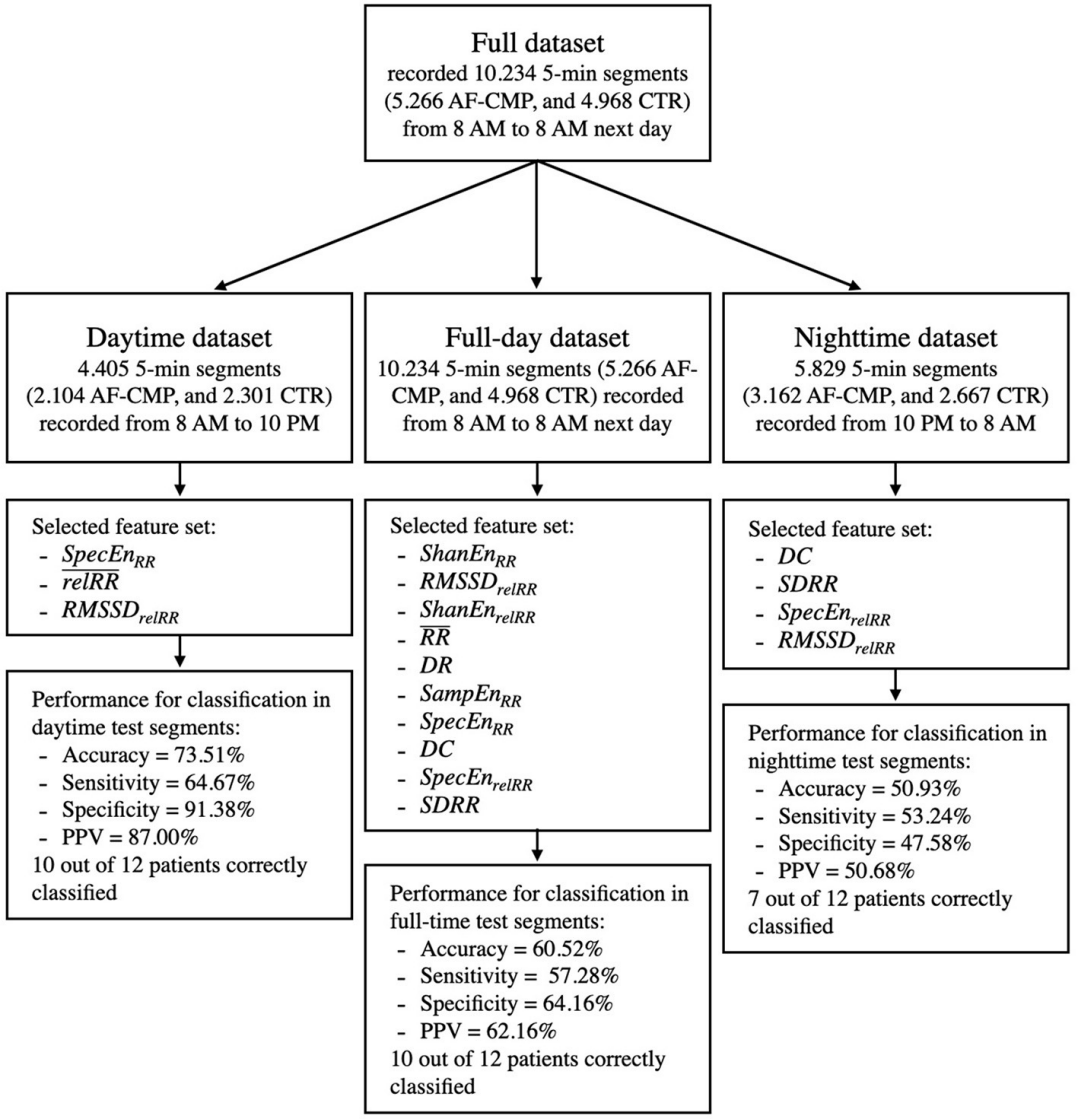
Overview of the decision tree classifier performance on the optimal feature sets selected from the different datasets utilized in this work. Control group (CTR).

Optimization for segments recorded during nighttime (10 p.m. to 8 a.m.) led to a feature set that comprised four features out of the 14 extracted features (*DC*, *SDRR*, *SpecEn*_*relRR*_, and *RMSSD*_*relRR*_). The classifier retrained on this optimized feature set yielded a nighttime test set accuracy of 50.4%, specificity of 47.6%, sensitivity of 53.2%, and PPV of 50.7%, and 7/12 patients (58.3%) were classified correctly (3/6 control group patients, and 4/6 AF-HF patients). The difference in accuracy between the three different classifiers is visually shown in the Supplementary Material, whereas an overview of the performance that the decision tree classifier achieved in the different datasets using the respective feature sets is shown in Figure 4.

## Discussion

The current study reports three main findings: First, patients with AF-HF differ from control group patients without heart failure with regard to heartbeat entropy (*SpecEn*_*RR*_) and beat-to-beat variation ($\bar{relRR}$ and *RMSSD*_*relRR*_) during AF. Second, incorporation of these individual features in a machine learning algorithm correctly stratifies the majority of test patients to AF-HF or control group. And third, circadian analysis of algorithm performance demonstrates superior discriminative properties during daytime.

### Heart Rate in AF and Development of LVSD–The Fast and the Furious?

Epidemiological studies demonstrate that heart failure and AF predispose to each other, and often co-exist (13). AF may worsen heart failure symptoms in patients with various underlying cardiomyopathies such as ischemic or valvular heart disease (“AF-associated” cardiomyopathy) or serve as the only causative reason for LVSD (AF-HF). The pathophysiology of AF-HF is not entirely understood, and proposed mechanisms include immunological alterations (14) as well as abnormalities in energy metabolism or calcium handling (15).

Rapid ventricular heart rates during AF are often being associated with AF-HF. As such, rapid atrial pacing is a common model to induce LVSD in animals, and heart rate control was shown to be non-inferior to rhythm control in older heart failure trials (16). However, average heart rates below 100 bpm in AF may equally lead to severe forms of AF-HF (6), demonstrating that heart rate alone is likely not a suitable discriminator for AF-HF in clinical practice.

In the current study, we investigated various features that describe entropy, variability but also beat-to-beat heart rate in patients with AF-HF. The most important features for discrimination of patients with AF-HF from control group patients were all related to entropy and variability (*SpecEn*_*RR*_, *RMSSD*_*relRR*_, and $\bar{relRR}$). This finding is in line with the clinical observation that arrhythmia-induced heart failure occurs not only in the context of chronic tachycardia but also with frequent premature atrial or ventricular contractions (15).

### Machine-Learning for Patient Stratification

For the current study, fourteen features commonly used for the analysis of heart rate variability and regularity were extracted from 5-min RR-series segments. The 5-min intervals were chosen following the recommendation given by the European Society of Cardiology and the North American Society of Pacing and Electrophysiology regarding the standardization of physiological and clinical studies (8). The decision tree classifiers for binary classification of AF-HF vs. control group achieved a clinically useful specificity and positive predictive value of 91.4 and 87.0%, respectively, using only three features (*SpecEn*_*RR*_, $\bar{relRR}$, and *RMSSD*_*relRR*_). The most important contribution to the algorithm's performance was given by *SpecEn*_*RR*_ (Figure 2), with lower *SpecEn*_*RR*_ values corresponding to decreased spectral complexity (the number of frequencies of which the signal is composed) in patients with AF-HF.

Remarkably, the abovementioned features that were automatically selected for the classifier are relatively novel, and the scientific literature reporting on their application in patients with AF is scarce. In this context, spectral entropy was previously shown to predict outcomes in AF patients, and to discriminate between persistent, and long-standing AF (17). In patients with sinus rhythm, analysis of spectral entropy was successfully used to discriminate healthy patients from patients with heart failure (18). In line with our findings, heart failure in this study was associated with lower a spectral entropy.

$\bar{relRR}$ has been proposed as a robust, simple, and reliable measure of heart rate variability, aiming to overcome the shortcomings of conventional measures of HRV, with *RMSSD*_*relRR*_ being a direct derivative (19). $\bar{relRR}$ was successfully applied in machine learning algorithms to differentiate atrial fibrillation from sinus rhythm (20). To our knowledge, the current study is the first clinical evaluation of the performance of these parameters for stratification of AF-induced heart failure.

In contrast to the good performance of the algorithm when derived from and applied to RR-intervals recorded during the day, application of the algorithm to data recorded at nighttime performed significantly worse even after optimization of the feature set. It is possible that influences of factors such as physical activity, or autonomic nervous tone, and the concentration of catecholamines in serum are pronounced during the day and blunted at night, although the current study does not allow to draw causative relations in this context.

## Future Perspective

Current clinical guidelines (1) emphasize the association of heart failure, and AF both during the initial diagnostic workup for new-onset AF, as well as during follow-up: they request a baseline echocardiogram in patients with new-onset AF, and they recommend regular clinical follow-up for the development of heart failure in patients with known AF. The algorithm reported in the current study may be particularly useful for the latter part, i.e., detection of LVSD in patients with AF. Due to its high specificity, and positive predictive value, it can act as an indicator, and trigger for prompt clinical follow-up to detect, and manage heart failure early, and potentially reduce mortality (21). In this context, the general applicability of the algorithm to all kinds of 5-min samples of RR intervals without the need for more than one lead (such as data derived from pulse wave analysis, oximetry derived heart rate or single-lead smart watch recordings) might enable the translation to a variety of wearables, and pocket ECG monitors.

## Limitations

The current study was restricted to the analysis of beat-to-beat intervals that were extracted from a single-lead ECG. This approach is however potentially also applicable to any device offering beat-to-beat annotations of the cardiac cycle, which may include widely applicable devices such as e.g., photo-plethysmography in smart phones although this will require additional validation. While the performance of the current algorithm is superior when applied on daytime-datasets, the impact of varying physiological conditions during daytime such as physical activity or mental stress, is beyond the scope of the current study. Also, we cannot comment on the influence of pertinent baseline medications on the performance of the current algorithm, though its applicability on a real-world patient cohort likely adds to its external validity.

## Conclusion

The current work demonstrates that machine learning with the simple input of beat-to-beat intervals from a single-lead ECG allows discriminating AF patients with, and without AF-induced heart failure with diagnostic properties that are immediately clinically applicable. Given the ever-increasing prevalence of AF, the algorithm described in this study may allow to identify patients who require cardiological care earlier and render the clinical follow-up more cost-effective.

## Data Availability Statement

The raw data supporting the conclusions of this article will be made available by the authors, without undue reservation.

## Ethics Statement

The studies involving human participants were reviewed and approved by Institutional Review Board of the University of Freiburg. The patients/participants provided their written informed consent to participate in this study.

## Author Contributions

FR, CA, LM, F-JN, TA, AJ, and BM-E were involved in data collection. GL, DN, MR, OD, RS, and AL were involved in the machine learning application. GL and BM-E wrote the initial manuscript. All authors read, reviewed, and edited the manuscript in the subsequent revision rounds.

## Funding

This work was supported by the European Union's Horizon 2020 research and innovation program under the Marie Sklodowska-Curie [Grant agreement no. 766082, MY-ATRIA project]. The funders were not involved in the design and execution of this study. We gratefully acknowledge financial support by the (DFG) through project number 183027722.

## Conflict of Interest

The authors declare that the research was conducted in the absence of any commercial or financial relationships that could be construed as a potential conflict of interest.

## Publisher's Note

All claims expressed in this article are solely those of the authors and do not necessarily represent those of their affiliated organizations, or those of the publisher, the editors and the reviewers. Any product that may be evaluated in this article, or claim that may be made by its manufacturer, is not guaranteed or endorsed by the publisher.
