# Supplementary material for: Machine Learning Using a Single-Lead ECG to Identify Patients With Atrial Fibrillation-Induced Heart Failure
Source: Front Cardiovasc Med. 2022 Feb 28;9:812719. doi: 10.3389/fcvm.2022.812719 (PMC8918925; doi:10.3389/fcvm.2022.812719)
Supplement: Supplementary file 1 [file Data_Sheet_1.docx]

Supplementary Material

# Feature extraction

Table 1. List of extracted features.

| 1.$\bar{RR}$ | 2. $\bar{relRR}$ | 3. $SDRR$ | 4.${SDRR}_{rel}$ | 5.${RMSSD}_{RR}$ |
| --- | --- | --- | --- | --- |
| 6. ${RMSSD}_{relRR}$ | 7. $DC$ | 8.$DR$ | 9.${ShanEn}_{RR}$ | 10.${ShanEn}_{relRR}$ |
| 11. ${SampEn}_{RR}$ | 12. ${SampEn}_{relRR}$ | 13. ${SpecEn}_{RR}$ | 14.${SpecEn}_{relRR}$ |  |

## Beat-to-beat variability metrics

The mean value, the standard deviation, and the root mean square successive difference ($RMSSD$) of RR and relRR signals are very common clinical metrics that can easily represent the beat-to-beat variance in heart rhythm (1-2). The $RMSSD$ is a time domain measure of heart period variability in heart period series. All these features were calculated for both RR and relRR signals (feat. 1-6 in Table 1).

## Deceleration capacity and reserve

Deceleration capacity ($DC$) is computed through the Phase-Rectified Signal Averaging technique (PRSA) introduced by Bauer et al. (3).which detects and quantifies quasi-periodic oscillations masked by non-periodic components, and artifacts. $DC$ aims to provide a measure of cardiac vagal modulations. Following Bauer’s approach, the hyperparameters of the methods T and s have been set to 1 and 2, respectively.

Deceleration reserve ($DR$) emphasizes asymmetric growing and decaying heart rate trends and non-stationarity. This parameter is calculated as the sum of the deceleration capacity and the acceleration capacity (parameter similar to the $DC$, and it is calculated with the PRSA as well). $DC$ and $DR$ were calculated on the RR signals according to Rivolta et al. (4) (feat. 7-8 in Table 1).

## Entropy features

The Shannon entropy ($ShanEn$) is the measure of the uncertainty of occurrence of certain event, or information inherent in the signals’ possible outcomes (5). The $ShanEn$ was calculated for the RR and the relRR signals, and the features extracted were the ${ShanEn}_{RR}$, and ${ShanEn}_{relRR}$ (feat. 9-10 in Table 1).

Sample entropy ($SampEn$) is a measure of complexity that can be easily applied to any type of time series data, including heart rate variability. $SampEn$ is conceptually similar to approximating entropy, but it does not count self-matching, and it depends less on the data size. This property makes it amenable to applications with relatively short data size (6). The $SampEn$ was calculated for the RR and the relRR signals, and the features extracted were the ${SampEn}_{RR}$, and ${SampEn}_{relRR}$ (feat. 11-12 in Table 1). The embedding dimension parameter, m, was set to 1, and the tolerance parameter, r, was set to 0.2 (percentage applied to the SD).

The spectral entropy ($SpecEn$) of a signal is a measure of its spectral power distribution. The concept is based on the Shannon entropy, or information entropy, in information theory. The $SpecEn$ treats the signal’s normalized power distribution in the frequency domain as a probability distribution, and calculates the Shannon entropy of it. The Shannon entropy in this context is the spectral entropy of the signal (7). The $SpecEn$ was calculated for the RR and the relRR signals with the MATLAB *pentropy*. The features extracted were the ${SpecEn}_{RR}$, and ${SpecEn}_{relRR}$ (feat. 13-14 in Table 1). In Supplementary Figure 1 the ${SpecEn}_{RR}$ distribution for the CTR and AF-HF groups is reported due to the relevance that this feature showed to perform the developed classification.


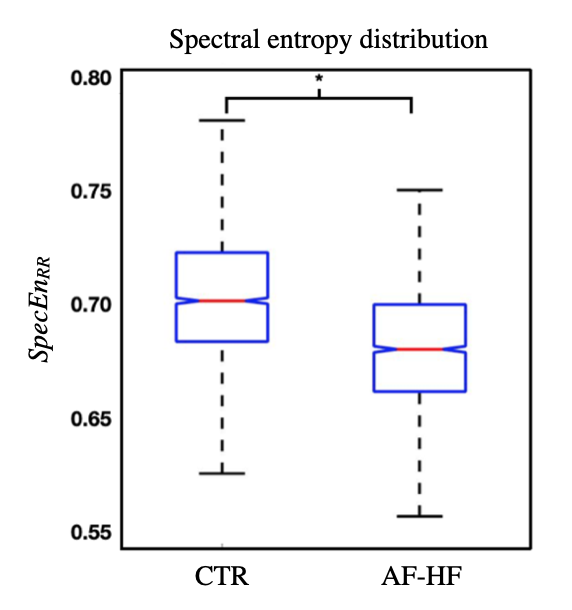


Supplementary Figure 1. Comparison of the $\boldsymbol{SpecEn}_{\boldsymbol{RR}}$ values’ distribution for the classification among CTR and AF-HF groups.

# Patient’s test set accuracy

Table 2. Number of segments and accuracy for each individual patient in the test set (%) for the full-day/night datasets, respectively. The last column shows which patients got correctly classified over the segments (+) and which were misclassified (-).

| **Test set patient ID** | **No. segments**  **(full-day/night)** | **Class** | **ACC_P_i_**  **(full-day/night)** | **Correct classification (full-day/night)** |
| --- | --- | --- | --- | --- |
| 1 | 186/114 | AF-HF | 54.64/72.73 | +/+ |
| 2 | 181/104 | AF-HF | 63.39/54.46 | +/+ |
| 3 | 190/105 | AF-HF | 58.03/41.28 | +/- |
| 4 | 170/106 | AF-HF | 68.00/52.38 | +/+ |
| 5 | 188/110 | AF-HF | 66.23/59.81 | +/+ |
| 6 | 206/107 | AF-HF | 30.46/37.62 | -/- |
| 7 | 177/92 | CTR | 65.82/60.75 | +/+ |
| 8 | 188/100 | CTR | 53.89/38.46 | +/- |
| 9 | 213/101 | CTR | 66.30/51.96 | +/+ |
| 10 | 184/106 | CTR | 71.75/55.05 | +/+ |
| 11 | 205/109 | CTR | 78.70/40.57 | +/- |
| 12 | 173/107 | CTR | 48.90/39.60 | -/- |

# Circadian classifiers’ accuracy

##
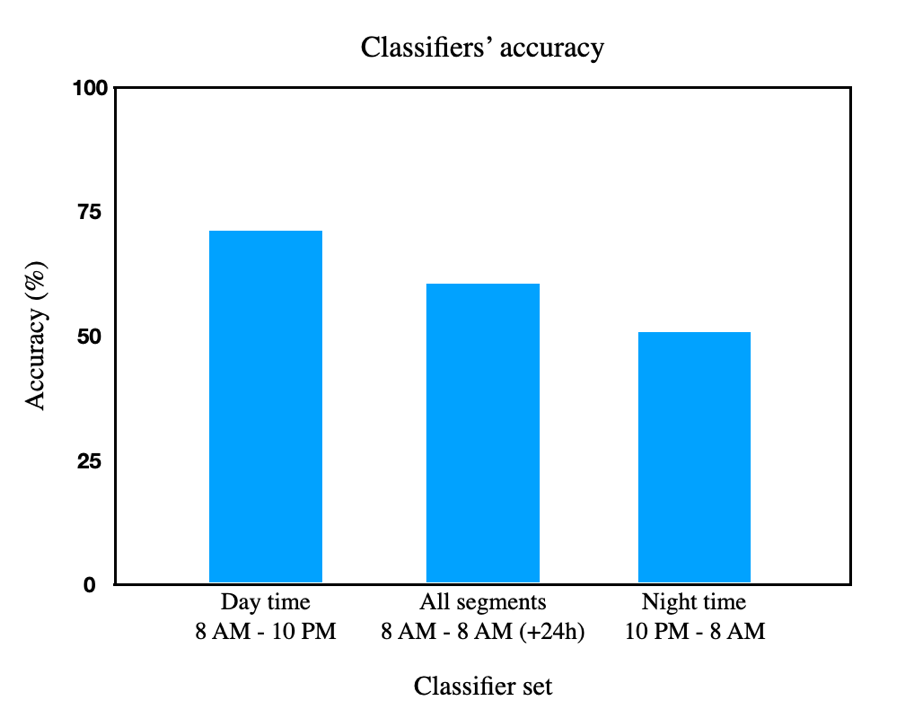


Supplementary Figure 2. Accuracy of the proposed classifiers for the Full-day, Day, and Night sets, respectively.

# Alternative machine learning algorithm approaches

In this study, we focused on the implementation of a decision tree classifier for the binary classification due to its simplicity and explainability. However, several machine learning algorithms have been tested (i.e., K-nearest-neighbor – KNN, and neural network -NN). The implementation and optimization of the classifiers followed the same procedure as described in the manuscript for the decision tree (section 2.5).

The KNN for the classification AF-HF vs. CTR patients using 5-minute RR segments achieved an accuracy of 72.2% on the test set with a sensitivity of 62.4%, a specificity of 91.6%, and a PPV of 86.0%. When applying a 50% threshold on the fraction of segments correctly classified for a given patient, 10 out 12 patients (83.3%) were correctly assigned to AF-HF or CTR (6/6 patients in the CTR group and 4/6 in the AF-HF group.

The NN for the classification AF-HF vs. CTR patients 5-minute RR segments achieved an accuracy of 75.1% on the test set with a sensitivity of 65.0%, a specificity of 89.9%, and a PPV of 85.4%. When applying a 50% threshold on the fraction of segments correctly classified for a given patient, 10 out 12 patients (83.3%) were correctly assigned to AF-HF or CTR (6/6 patients in the CTR group and 4/6 in the AF-HF group.

The above results showed that the decision tree classifier performed equally to the other two classification approaches.

# Study protocol and timepoints for patients’ admission


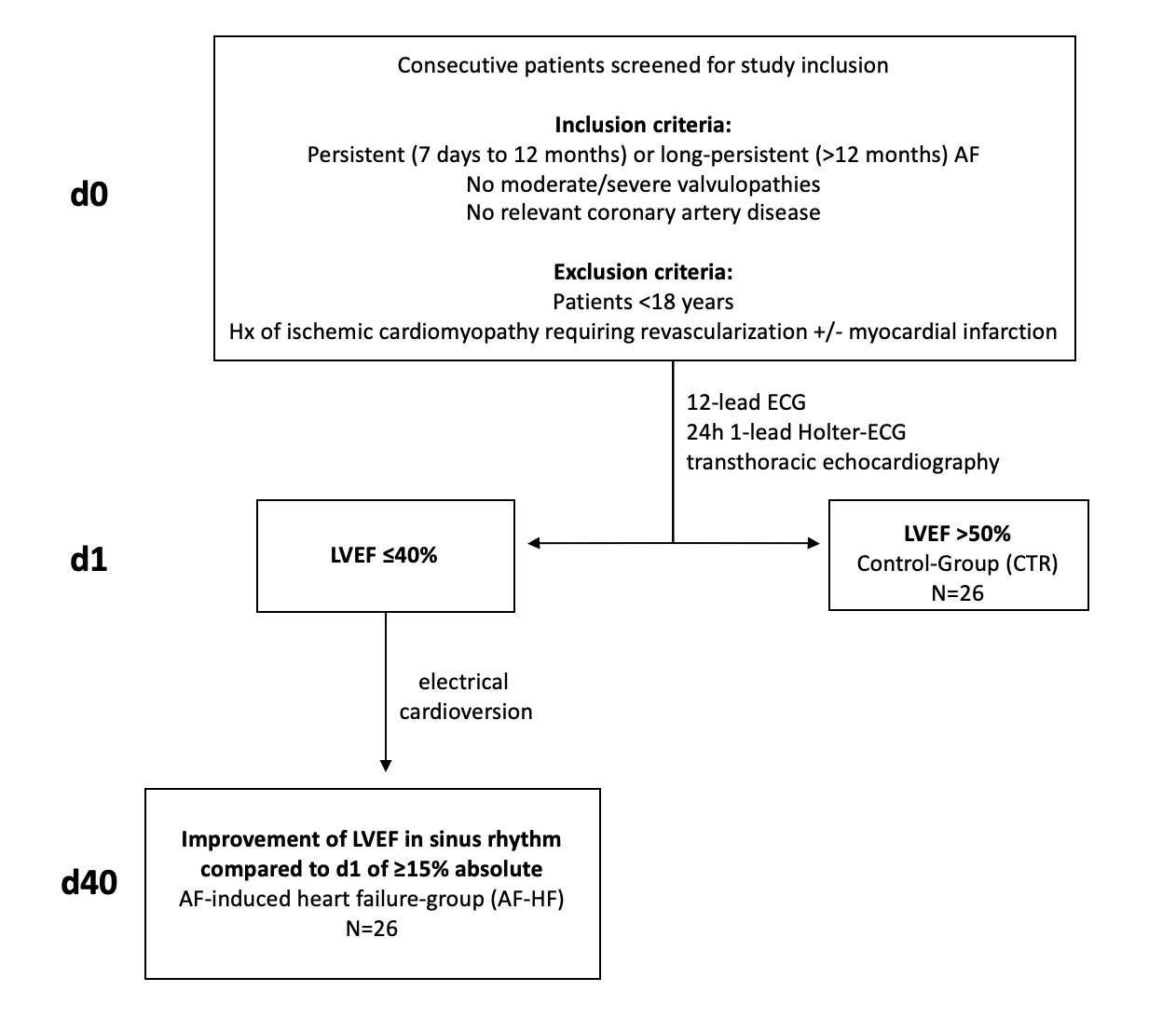


Supplementary Figure 3. Study protocol scheme for patients’ admission.

# Classification analysis with signals of different lengths

The analysis implemented in the main manuscript to classify between AF-HF and CTR patients was performed not only with 5-minute segments (as reported in the main manuscript) but also with 1-minute, 2.5-minute, and 15-minute segments so as to identify the best signal length needed to perform the automatic prediction.

Application of the decision tree classifier with the same feature set described in the main text on the patients in the test set from six AF-HF and six CTR patients yielded an overall accuracy to correctly assign a given 1-minute segment to AF-HF or CTR of 59.8%, with a specificity of 66.4%, sensitivity of 49.2%, and PPV of 67.3%. When applying a 50% threshold on the fraction of segments correctly classified for a given patient, 6 out 12 patients (50.0%) were correctly assigned to AF-HF or CTR (4/6 patients in the CTR group and 2/6 in the AF-HF group).

Application of the decision tree classifier with the same feature set described in the main text on the patients in the test set from six AF-HF and six CTR patients yielded an overall accuracy to correctly assign a given 2.5-minute segment to AF-HF or CTR of 60.2%, with a specificity of 73.4%, sensitivity of 60.8%, and PPV of 75.6%. When applying a 50% threshold on the fraction of segments correctly classified for a given patient, 8 out 12 patients (66.7%) were correctly assigned to AF-HF or CTR (4/6 patients in the CTR group and 4/6 in the AF-HF group).

Application of the decision tree classifier with the same feature set described in the main text on the patients in the test set from six AF-HF and six CTR patients yielded an overall accuracy to correctly assign a given 15-minute segment to AF-HF or CTR of 64.8%, with a specificity of 79.6%, sensitivity of 61.7%, and PPV of 78.2%. When applying a 50% threshold on the fraction of segments correctly classified for a given patient, 8 out 12 patients (66.7%) were correctly assigned to AF-HF or CTR (5/6 patients in the CTR group and 3/6 in the AF-HF group).

The classification with the 5-minute segments obtained better results than the other cases, confirming the reasons why it is recognized as conventional short-term recording standards by the international scientific community (Task Force of the European Society of Cardiology the North American Society of Pacing Electrophysiology 1996).

# Transthoracic echocardiography

Echocardiography was performed according recent recommendations (8). Left ventricular diameters were obtained by M-Mode, left ventricular and left atrial volumina by biplane modified Simpson’s method. In atrial fibrillation mean values of 5 representative beats were documented. Degree of mitral regurgitation was assessed visually.

# References

1. F. Shaffer and J. P. Ginsberg, “An Overview of Heart Rate Variability Metrics and Norms,” *Front. Public Heal*., 2017;5:258. doi: 10.3389/fpubh.2017.00258.
2. M. Vollmer, "A robust, simple and reliable measure of heart rate variability using relative RR intervals," *2015 Computing in Cardiology Conference (CinC)*, 2015,609-612, doi: 10.1109/CIC.2015.7410984.
3. Bauer, J. W. Kantelhardt, A. Bunde, et al., “Phase-rectified signal averaging detects quasi-periodicities in non-stationary data,” *Physica A*, 2006;364:423–434.
4. M. W. Rivolta, T. Stampalija, M. G. Frasch, and R. Sassi, “Theoretical Value of Deceleration Capacity Points to Deceleration Reserve of Fetal Heart Rate,” *IEEE Trans. Biomed. Eng*., 2020; 67(4):1176–1185. doi: 10.1109/TBME.2019.2932808.
5. C.E. Shannon, A mathematical theory of communication, The Bell System Technical Journal 1948;27(4):623-656. doi: 10.1002/j.1538-7305.1948.tb00917.x
6. Kijoon Lee (2021). Sample Entropy, MATLAB Central File Exchange. Retrieved October 13, 2021. <https://www.mathworks.com/matlabcentral/fileexchange/35784-sample-entropy>.
7. Vakkuri, A. Yli-Hankala, P. Talja, et al., Time-frequency balanced spectral entropy as a measure of anesthetic drug effect in central nervous system during sevoflurane, propofol, and thiopental anesthesia, *Acta Anaesthesiologica Scandinavica* 2004;48(2):145–153. doi:10.1111/j.0001-5172.2004.00323.x
8. R.M Lang, L.P. Badano, V. Mor-Avi et al., Recommendations for Cardiac Chamber Quantification, *J Am Soc Echocardiogr* 2015; 28:1.
